# Supplementary material for: Long-read sequencing of extrachromosomal circular DNA and genome assembly of a Solanum lycopersicum breeding line revealed active LTR retrotransposons originating from S. Peruvianum L. introgressions
Source: BMC Genomics. 2024 Apr 24;25:404. doi: 10.1186/s12864-024-10314-1 (PMC11044480; doi:10.1186/s12864-024-10314-1)
Supplement: Supplementary file 2 — Supplementary Material 2 [file 12864_2024_10314_MOESM2_ESM.docx]

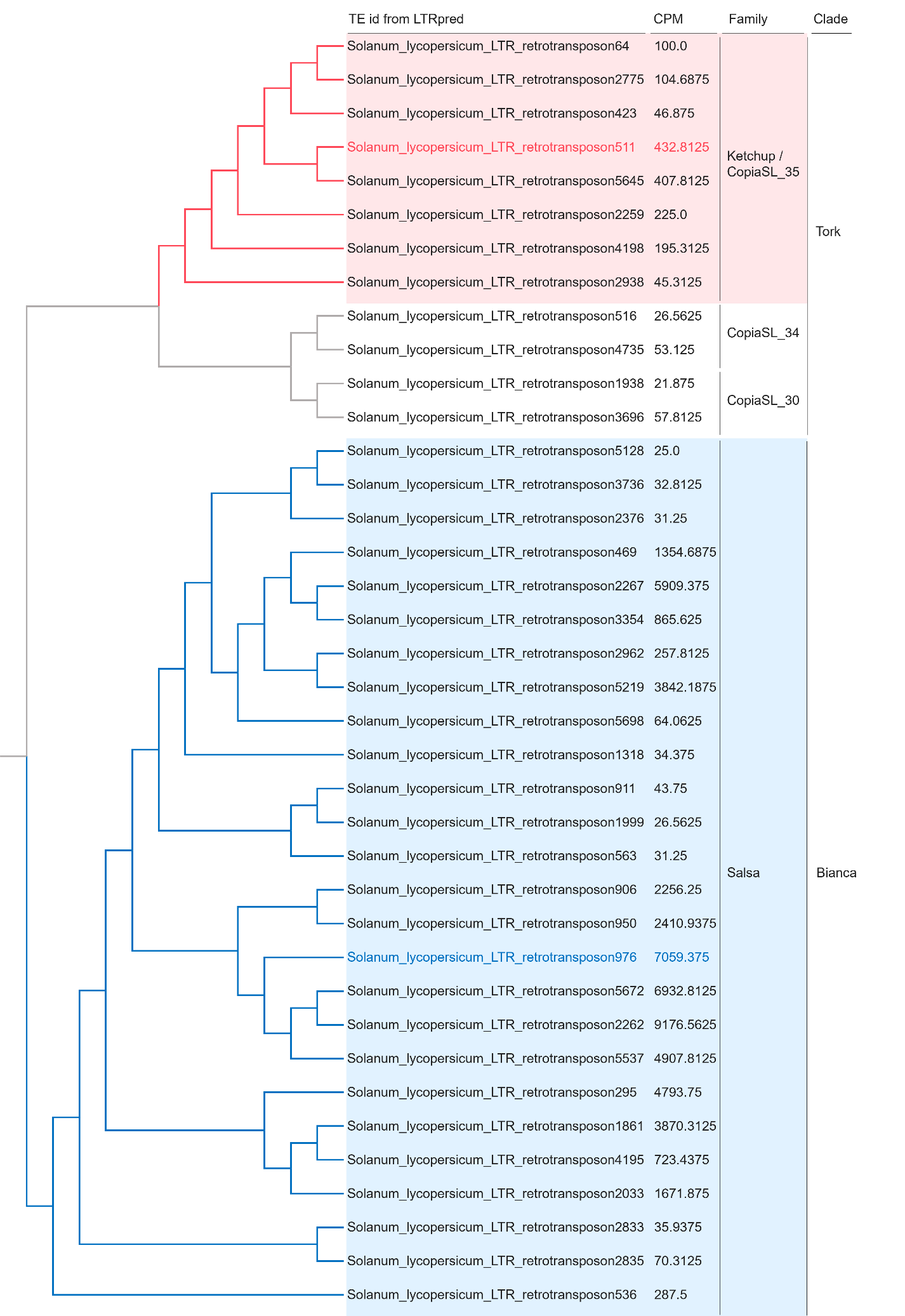


**Figure S1**. Phylogenetic tree of full-length sequences of 38 RTEs. The red and blue colors correspond to the elements of the *Ketchup* and *Salsa* families, respectively.


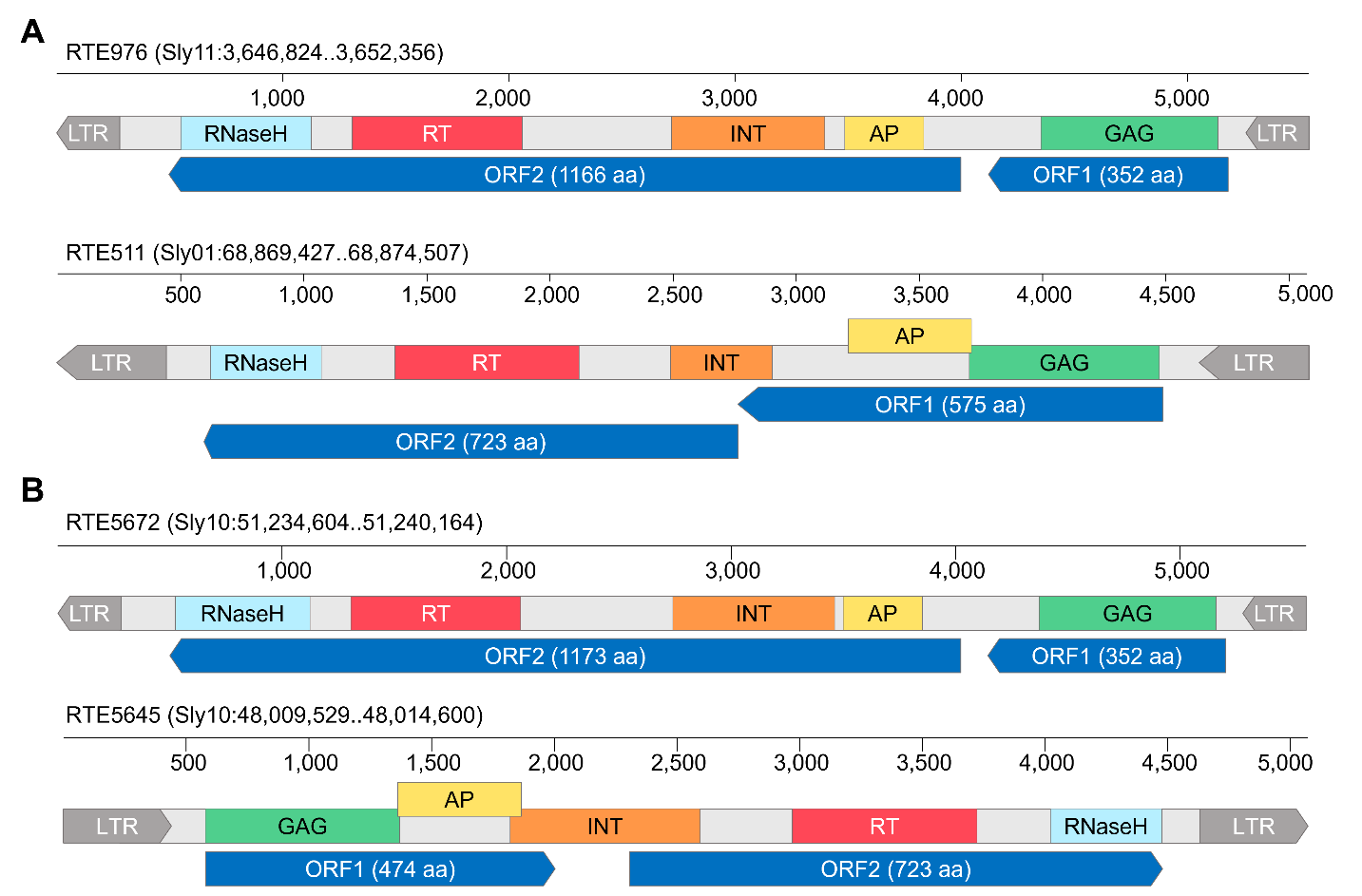


**Figure S2**. Encoded domains and open reading frames (ORFs) for RTEs (**A**) RTE976 and RTE511, two SL elements belonging to the *Salsa* and *Ketchup* family respectively; (**B**) RTE5672 and RTE5645, two SL elements belonging to the *Salsa* and *Ketchup* family respectively.


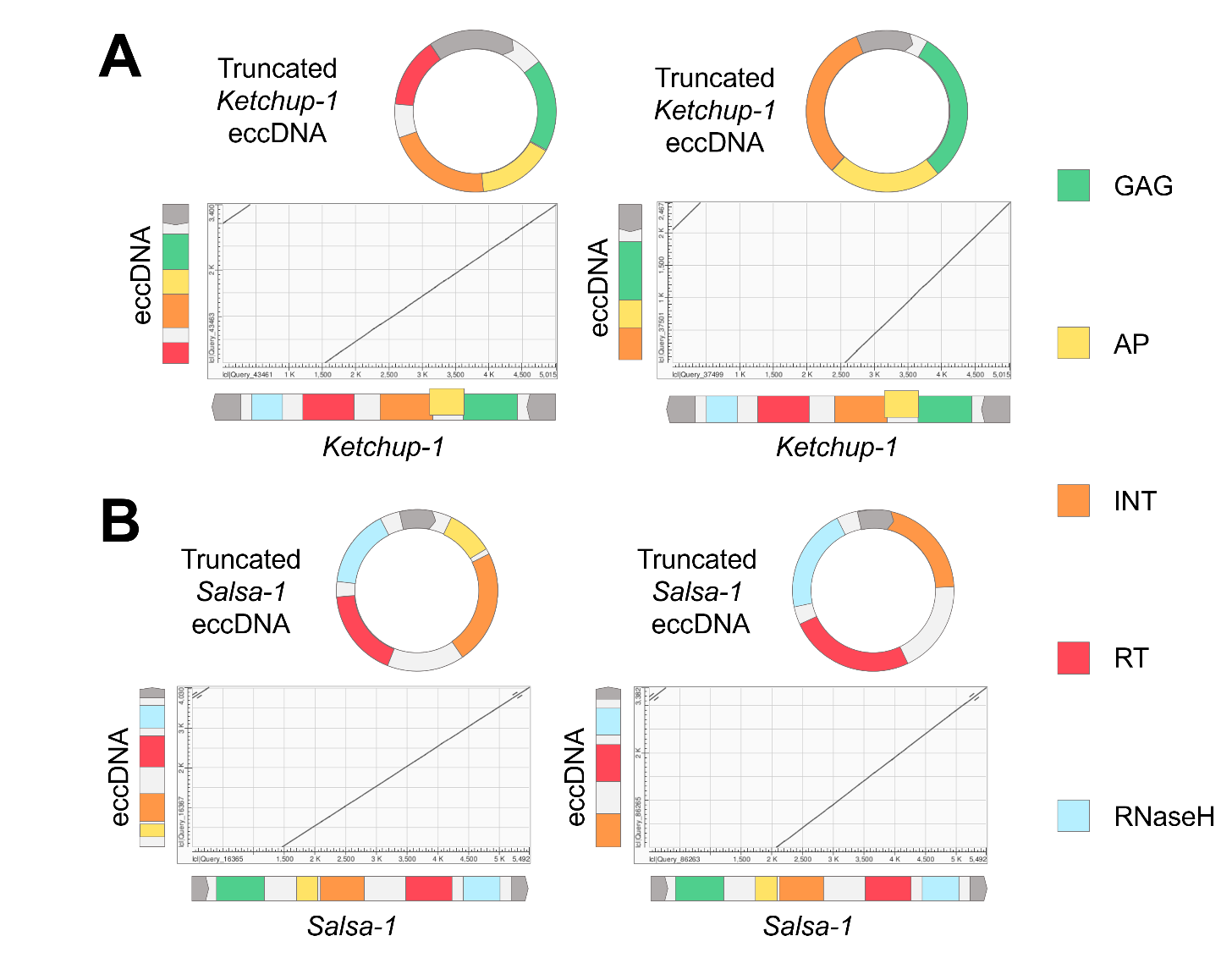


**Figure S3**. Dot plots for alignment of truncated eccDNA sequences (Y axis) and full-length retrotransposon sequences (X axis) with domain annotation: (**A**) – *Ketchup-1*; (**B**) – *Salsa-1*.


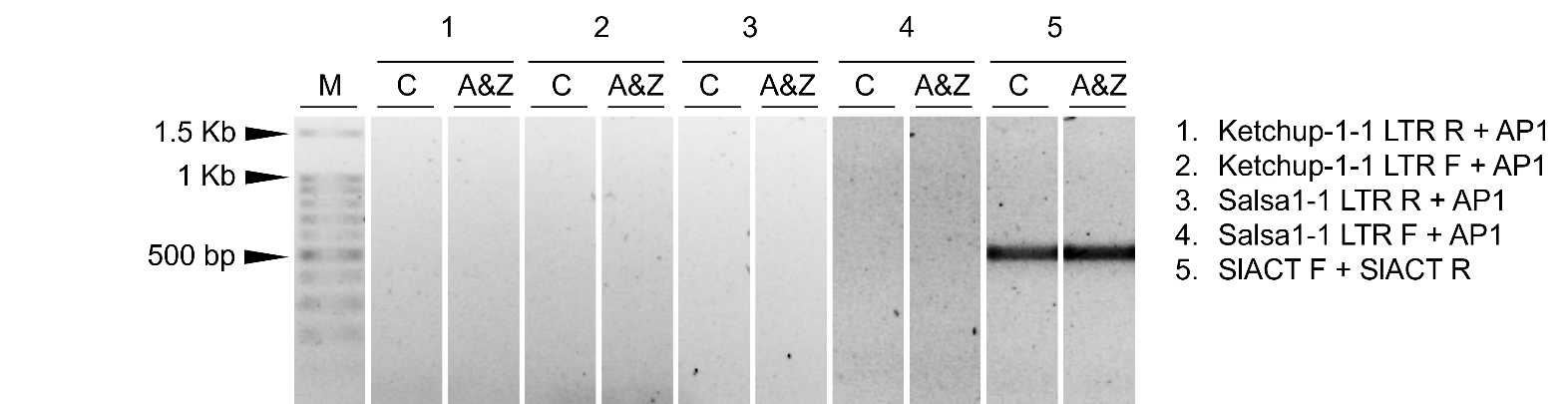


**Figure S4**. PCR confirmation of the absence of eclDNA accumulation for *Ketchup-1-1* and *Salsa-1-1* elements in control (C) and relaxed TE control (A&Z) samples.


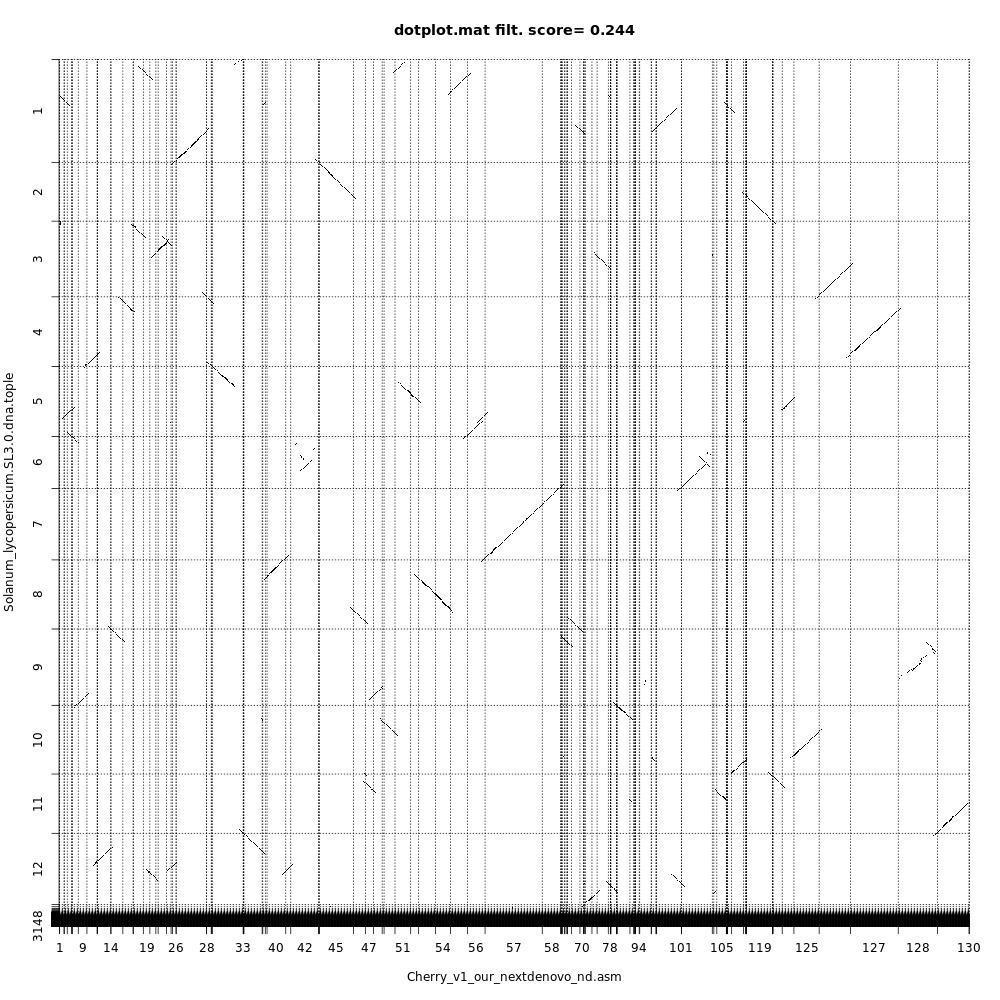


**Figure S5**. Dot plot for alignment of draft assembly contigs (X axis) and SL3.0 chromosomes (Y axis).


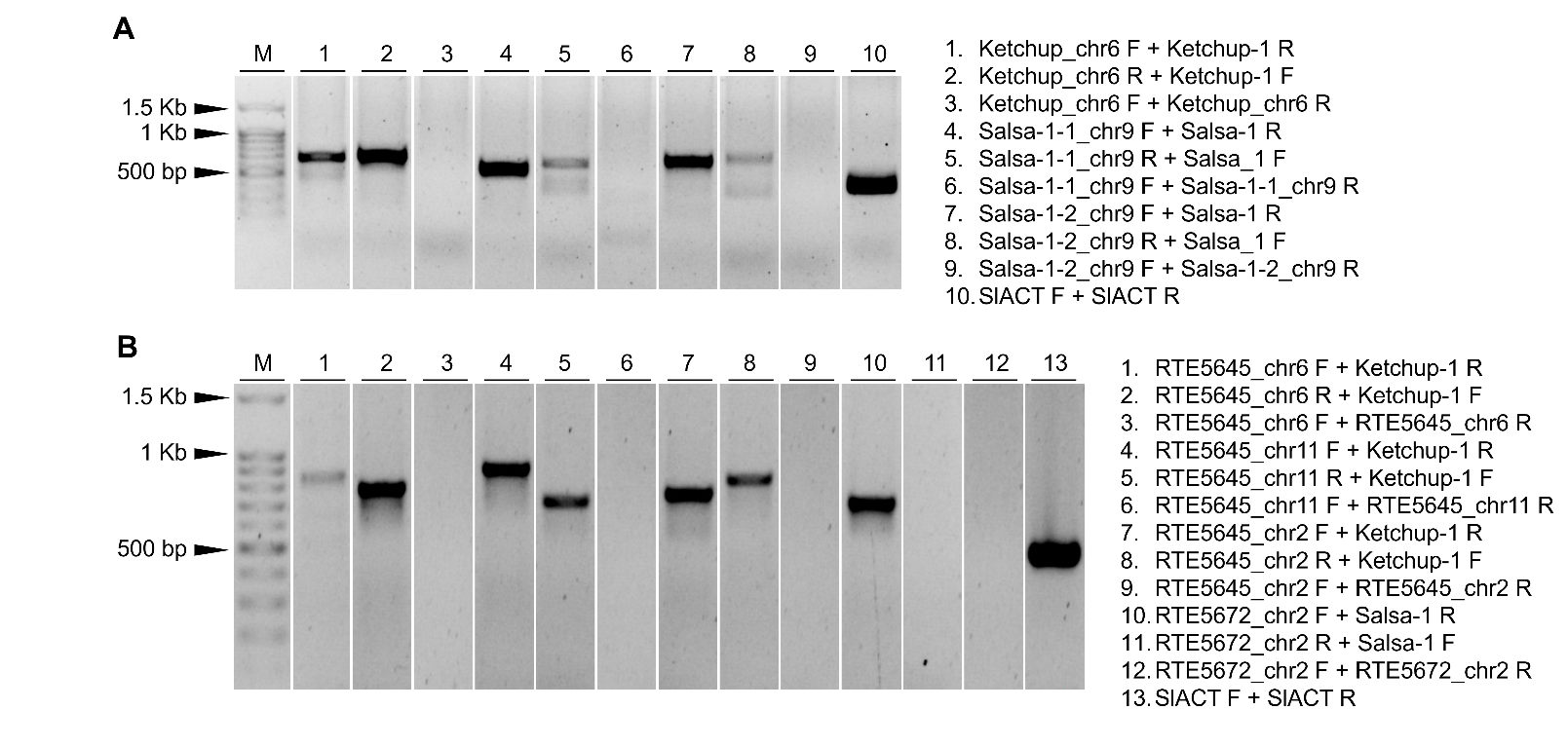


**Figure S6**. PCR validation of insertions of SP **(A**) and SL (**B**) *Ketchup and Salsa* elements TEIs in our tomato line.


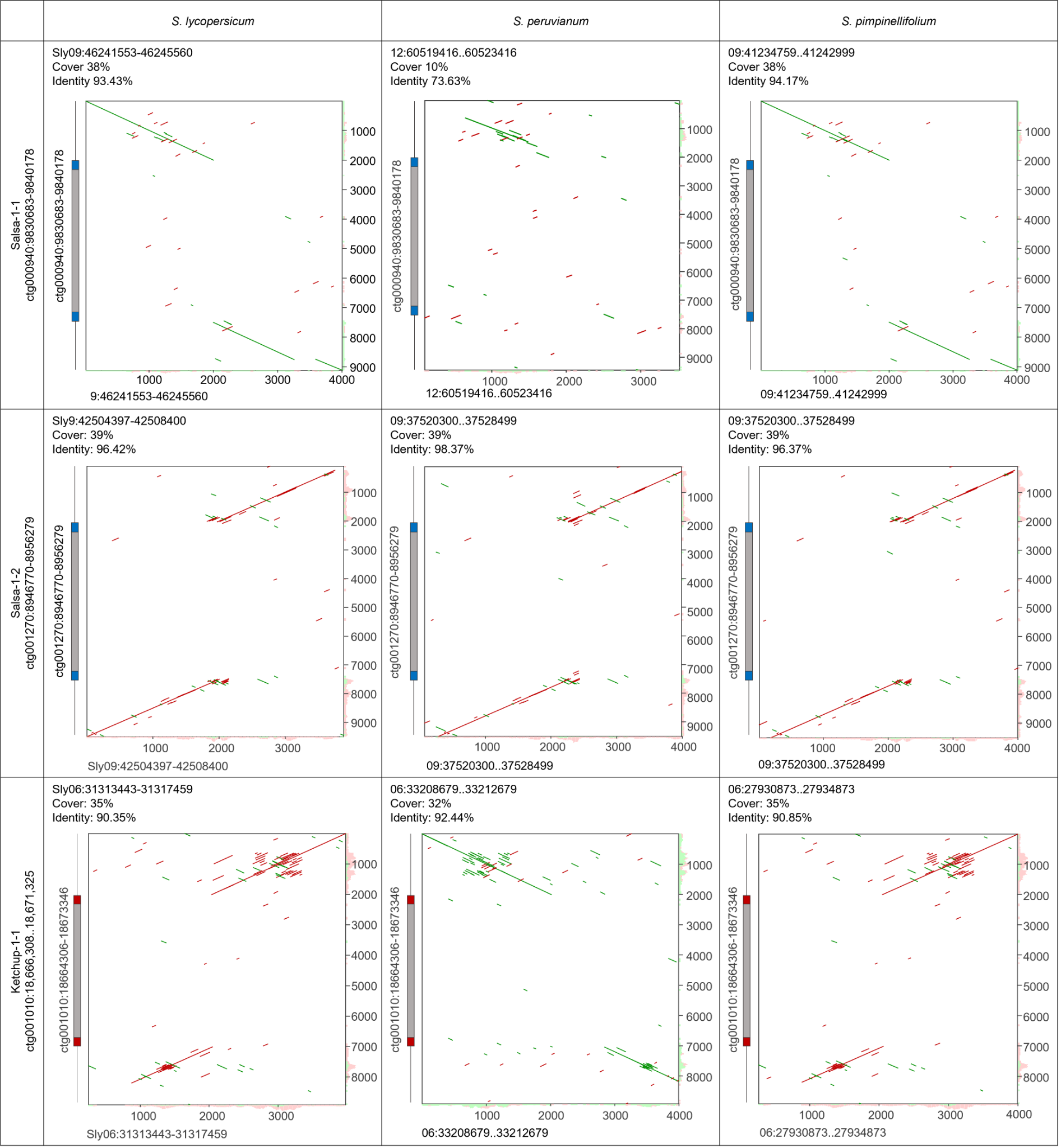
**Figure S7**. Dot plots for sequence alignments of introgressed TEIs with flanking regions with the corresponding regions of S. lycopersicum, S. peruvianum and S.pimpinellifolium genomes.
